# Transmission of a bumblebee parasite is robust despite parasite exposure to extreme temperatures

**DOI:** 10.1002/ece3.10379

**Published:** 2023-07-25

**Authors:** Hannah S. Wolmuth‐Gordon, Mark J. F. Brown

**Affiliations:** ^1^ School of Life Sciences and the Environment Royal Holloway University of London Egham UK

**Keywords:** climate, climate change, environmental exposure, epidemiology, global warming, host, infectivity, pollinator

## Abstract

All organisms are exposed to fluctuating environmental conditions, such as temperature. How individuals respond to temperature affects their interactions with one another. Changes to the interaction between parasites and their hosts can have a large effect on disease dynamics. The gut parasite, *Crithidia bombi*, can be highly prevalent in the bumblebee, *Bombus terrestris*, and is an established epidemiological model. The parasite is transmitted between bumblebees via flowers, exposing it to a range of environmental temperatures prior to infection. We investigated whether incubation duration and temperature exposure, prior to infection, affects parasite infectivity. Prior to inoculation in *B. terrestris*, *C. bombi* was incubated at 10, 20, 30, 40 or 50°C for either 10 or 60 min. These times were chosen to reflect the length of time that the parasite remains infective when outside the host and the rate of floral visitation in bumblebees. Prevalence and infection intensity were measured in bees 1 week later. Incubation duration and the interaction between incubation temperature and duration affected the prevalence of *C. bombi* at 50°C, resulting in no infections after 60 min. Below 50°C, *C. bombi* prevalence was not affected by incubation temperature or duration. Extreme temperatures induced morphological changes in *C. bombi* cells; however, infection intensity was not affected by incubation duration or temperature. These results highlight that this parasite is robust to a wide range of temperatures. The parasite was not infective after being exposed to 50°C for 60 min, such temperatures likely exceed the flight abilities of bumblebees, and thus the potential for transmission. This study shows the importance of understanding the effects of environmental conditions on both hosts and parasites, which is needed to predict transmission under different environmental conditions.

## Graphical abstract

The transmission of a prevalent bumblebee gut parasite is robust to exposure to extreme temperatures. Therefore, its transmission is unlikely to be affected by temperature differences across the seasons, between climates and with climate change in the future.

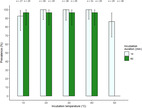

## 1 INTRODUCTION

To survive, all organisms must respond to changing environments. One variable environmental condition is temperature, which fluctuates substantially over a wide range of scales. For example, temperature can vary daily, seasonally, and across the year. In addition, mean temperatures are predicted to increase due to climate change in the coming decades (Wuebbles et al., 2017), posing a further challenge to organisms. All living organisms are sensitive to changes in temperature, and how individuals respond to these changes may affect their interactions with other species (Boukal et al., 2019; Doney et al., 2011). For host–parasite interactions, temperature can have large impact on disease dynamics (Altizer & Oberhauser, 1999; Frid & Myers, 2002; Menti et al., 2000). Consequently, understanding how temperature affects these interactions is necessary for predicting disease dynamics across seasons, in different climates and with climate change.

Temperature can affect disease dynamics directly through altering host susceptibility to infection (Adamo & Lovett, 2011; Murdock et al., 2012). Less obviously, but arguably more importantly, temperature can also affect host susceptibility indirectly. Higher temperatures may result in increased host growth and in turn, host density, leading to higher rates of transmission (Burdon & Chilvers, 1982). Parasites can be exposed to varying temperatures both inside and outside a host, and these temperatures can affect their survival rate, development rate (Kalinda et al., 2017; Leathwick, 2013) and infectivity (O'Connor et al., 2006). Within a host, parasites that infect poikilotherms, such as arthropods, are exposed to wider fluctuations in temperature compared to parasites of homeothermic hosts. Such fluctuations have major impacts on many vector‐borne diseases, such as those transmitted by mosquitoes (Liu‐Helmersson et al., 2014). In bumblebees and honeybees, Temperature variation has been shown to correlate with parasite prevalence and infection intensity (Chen et al., 2012; Manlik et al., 2023; McMullan & Brown, 2005; Retschnig et al., 2017). For example, *Nosema cerenae* infection in honeybee workers has been shown to correlate negatively with temperature (Chen et al., 2012; Retschnig et al., 2017), but this relationship can change depending on other factors, such as seasonality and host genotype (Manlik et al., 2023). Similar relationships exist outside of invertebrate hosts, for example, amphibian chytrid fungus that generally exhibits higher prevalence and infection intensity at lower temperatures (Ellison et al., 2020; Raffel et al., 2015; Sonn et al., 2017).

When outside the host, parasites may be exposed to highly variable environmental conditions. Therefore, parasites with free‐living stages and indirect transmission modes are particularly vulnerable to environmental temperature changes due to their period in the external environment. For example, the survival and infectivity of free‐living nematode larvae of sheep are affected by environmental temperature, with the optimum temperature varying between species (Morgan & van Dijk, 2012; O'Connor et al., 2006). Temperature has also been shown to affect the survival and viability of viruses in air‐borne droplets (Chen, 2020; Prussin et al., 2018), faeces (Moe & Shirley, 1982) and in water sources (Nasser & Oman, 1999). The direction of temperature effects on viral survival and infectivity can vary between viruses and with other environmental conditions, such as humidity (Chen, 2020; Moe & Shirley, 1982; Prussin et al., 2018).

Many pollinator parasites, including *Vairimorpha apis*, *V. bombi*, and *Crithidia bombi* are transmitted indirectly via the shared use of flowers (Adler et al., 2018; Durrer & Schmid‐Hempel, 1994; Figueroa et al., 2019; Graystock et al., 2015; Pinilla‐Gallego et al., 2022). These parasites are deposited across the whole flower, for example on the petals and in the nectar, by workers who may defecate when they forage (Durrer & Schmid‐Hempel, 1994; Figueroa et al., 2019; Pinilla‐Gallego et al., 2022). The subsequent location of the parasite on the flower will determine its exposure to the ambient temperature. Parasite cells on the petals will exhibit higher temperature variation compared to those in the nectar due to both exposure to direct sunlight and the higher specific heat capacity of nectar, a liquid, compared to the petals, a solid. As one would expect, parasite survival can be higher when deposited inside the floral structure, in the corolla, compared on more exposed structures, such as the bract (Figueroa et al., 2019). Furthermore, the duration of time the parasite is exposed to the environment depends on the visitation rate of the flower by pollinators. Visitation rate varies between flowers and correlates with the rate of nectar replenishment (Stout et al., 1998). In some cases, the period between flower visits may be up to an hour (Stout & Goulson, 2001).

*Crithidia bombi* is a prevalent gut parasite of bumblebees and is a well‐established epidemiological model (Schmid‐Hempel et al., 2019). It is transmitted faecal‐orally between colonies via flowers (Adler et al., 2018; Durrer & Schmid‐Hempel, 1994; Figueroa et al., 2019; Pinilla‐Gallego et al., 2022) and within colonies through contact with infected individuals and with contaminated nest material (Otterstatter & Thomson, 2007; Sah et al., 2021). It has a wide distribution, spanning Europe (Rutrecht & Brown, 2008; Shykoff & Schmid‐Hempel, 1991; Votavová et al., 2022), Australasia (Felden et al., 2022), North and South America (Cordes et al., 2012; Fernández et al., 2020; Gallot‐Lavallée et al., 2016; Gillespie, 2010; Schmid‐Hempel et al., 2014). *Crithidia bombi* exhibits seasonal variability in prevalence, which peaks in June and July in the northern hemisphere (Graystock et al., 2020; Parsche & Lattorff, 2018; Popp et al., 2012). It is not known whether temperature differences across the seasons contribute to these changes. *Crithidia bombi* growth has been measured in vitro and peaks at 33.7–34.4°C. At temperatures above 37.9°C growth is inhibited (Palmer‐Young et al., 2018, 2021). Interestingly, the temperature of peak growth in vitro does not align with the temperature at which hosts exhibit peak infection intensities. Palmer‐Young et al. (2019) demonstrated that when the common Eastern bumblebee (*Bombus impatiens*) was incubated at 21°C infection intensities were 81% higher compared to at 37°C. When hosts were incubated at a slightly lower temperature, 29°C, Tobin et al. (2019) found no difference in the prevalence or infection intensity of *C. bombi* compared to those incubated at 21°C.

The effect of temperature on *C. bombi* during environmental exposure has not been investigated. We do know that the longer the parasite is outside the host the lower the probability of transmission (Schmid‐Hempel et al., 1999) and survival (Pinilla‐Gallego et al., 2022). Given the changes in *C. bombi* growth in vitro at different temperatures (Palmer‐Young et al., 2018, 2021), it is plausible that temperature exposure on flowers affects its transmission between colonies. Furthermore, temperature may affect within colony transmission of the parasite. Although bumblebees try to maintain a constant temperature within the colony, through fanning when it is hot (Weidenmüller et al., 2002) and incubating (O'Donnell & Foster, 2001) and building wax coverings when it is cold (Jones & Oldroyd, 2006), in reality, the temperature of the colony will fluctuate with environmental temperature and colony size (Crall et al., 2018; Vogt, 1986). In addition, colony temperature varies between colonies in different climates, particularly at the start of their lifecycle when the colony temperature is similar to the ambient temperature (Hasselrot, 1960).

To test whether temperature exposure affects *C. bombi* infectivity, we exposed *C. bombi* to 10, 20, 30, 40 and 50°C for either 10 or 60 min in vitro and inoculated *Bombus terrestris audax* hosts. One week later, host prevalence and infection intensity were measured. We expected prevalence and infection intensity to peak at 30°C in alignment with its peak growth in vitro (Palmer‐Young et al., 2018, 2021). We also predicted that infection would be impeded by higher temperatures, due to a reduction in growth in vitro above 37°C (Palmer‐Young et al., 2018, 2021) and an 81% decrease in infection intensity when bees were incubated at 37°C compared to 21°C (Palmer‐Young et al., 2019). Finally, we predicted that prevalence and infection intensity would be higher after *C. bombi* was incubated for 10 compared to 60 min (Schmid‐Hempel et al., 1999).

## 2 MATERIALS AND METHODS

### 2.1 Experimental organisms

Eight *B. terrestris audax* colonies, with 85–150 workers each, were ordered from Agralan. The colonies were kept at 27.8 (± 0.71)°C, ambient humidity (43.7 (±4.2)%) and under red light. The colonies were fed with honeybee‐collected pollen (Agralan) and sterile sugar solution (50% concentration) ad libitum. To ensure colonies were free of infection, 10 individuals per colony were screened for *C. bombi*, *Apicystis* spp. and *Vairimorpha bombi* by viewing their faeces using a phase contrast microscope (Nikon Eclipse 50i) at ×400 magnification (Rutrecht & Brown, 2008).

**FIGURE 1:**
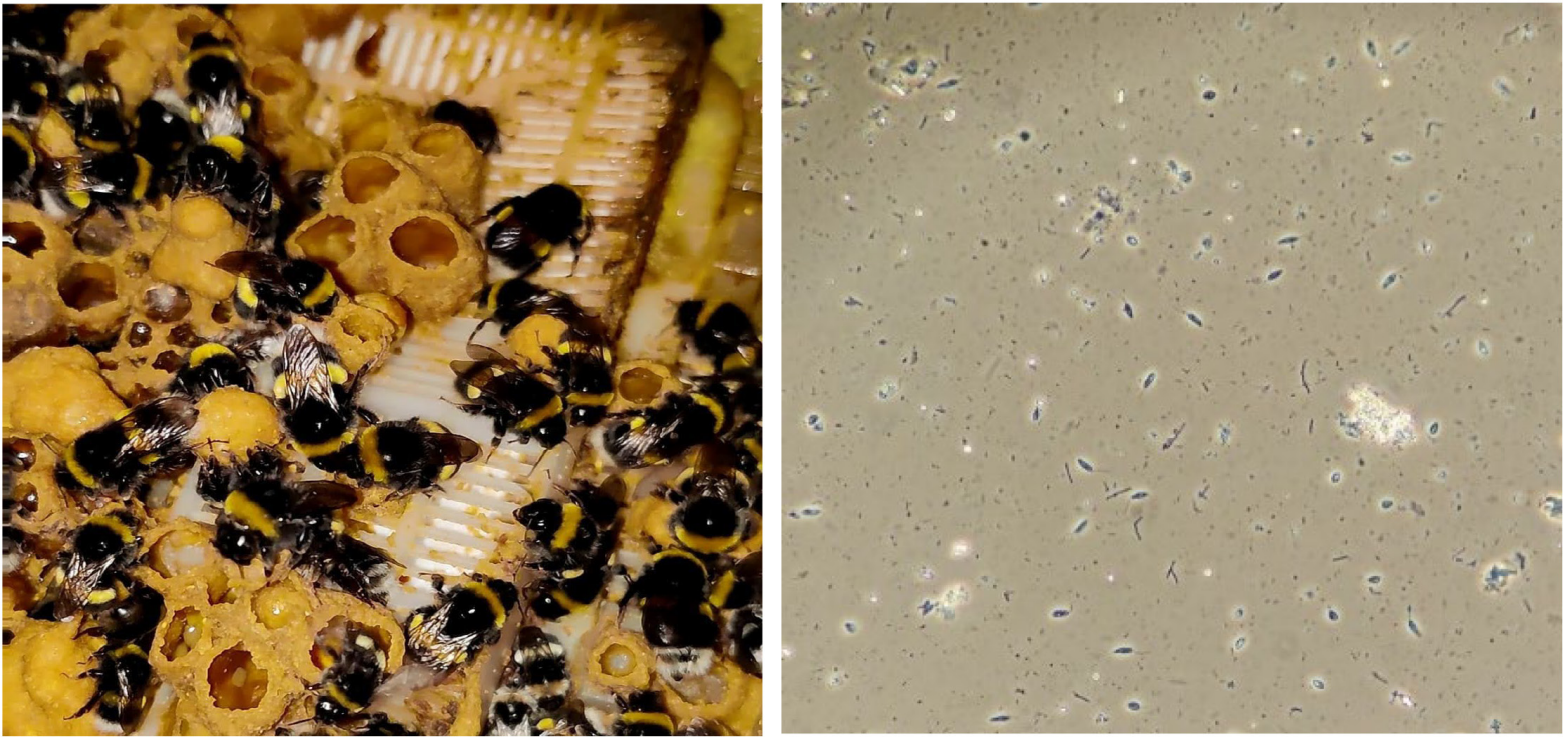
Photo of study species: Buff‐tailed bumblebee laboratory colony (*Bombus terrestris audax*, left) and *Crithidia bombi* at ×400 magnification under a phase contrast microscope (right).

*Crithidia bombi* was obtained from two laboratory stock colonies of *B. terrestris audax* (Agralan). The parasite was originally acquired from post‐hibernation spring queens of *B. terrestris audax* caught in Windsor Great Park (Surrey, UK) in March 2021, since when it has been continually cycled through laboratory colonies (Agralan). *Crithidia bombi* has three lifecycle stages. One lifecycle stage is non‐motile (amastigote) and two lifecycle stages are motile (choanomastigote and promastigote; Figure 1). The prevalence of each lifecycle stage can change over the course of an infection (Logan et al., 2005).

### 2.2 Experimental design

#### 2.2.1 Treatments

To find out whether temperature exposure prior to inoculation affected *C. bombi* infectivity, *C. bombi* was incubated at 10, 20, 30, 40 and 50°C in vitro. These temperatures were chosen after considering the temperatures at which bumblebees forage (Descamps et al., 2021; Heinrich, 1972; Kenna et al., 2021), the evaporation of nectar at higher temperatures (Descamps et al., 2021) and the growth of *C. bombi* at different temperatures in vitro (Palmer‐Young et al., 2018, 2021). *Crithidia bombi* was incubated at these temperatures for 10 and 60 min, in a fully factorial design. These incubation times were chosen based on the duration of time *C. bombi* remains infective (Schmid‐Hempel et al., 1999) and floral visitation rates of bumblebees in the field (Stout et al., 1998; Stout & Goulson, 2001). Our pilot experiments also indicated that these temperatures and incubation durations were suitable (results in Appendix S1).

#### 2.2.2 Counting the number of healthy and unhealthy *Crithidia bombi* cells in the inoculum prior to inoculation

**FIGURE 2:**
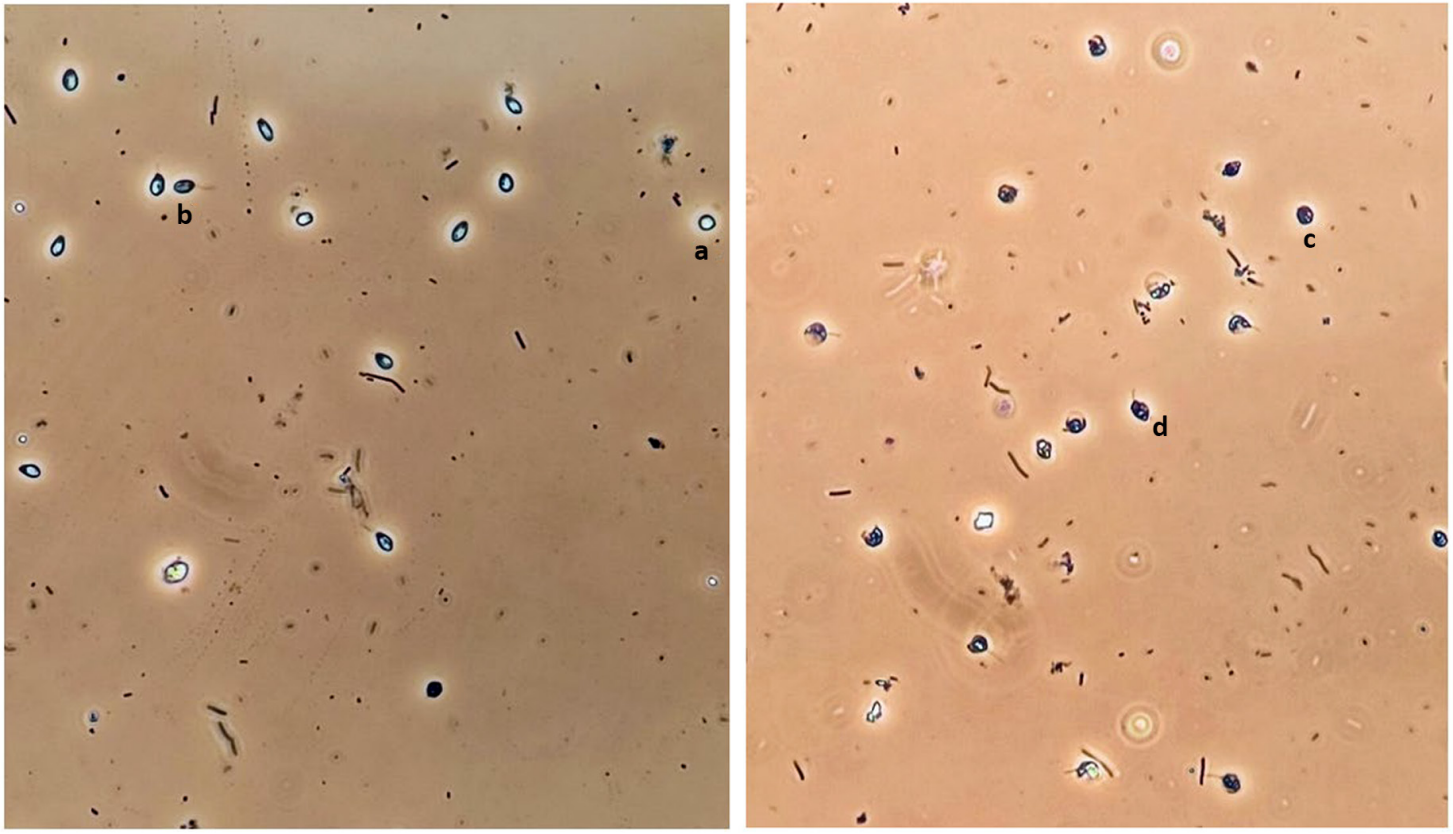
Photograph of *Crithidia bombi* cells at ×400 magnification using a phase contrast microscope. *Crithidia bombi* was suspended in 25% Ringer solution to aid photography. The left photograph shows *C. bombi* following incubation at 30°C for 60 min, which exhibits a large proportion of ‘healthy’ cells (a = healthy amastigote, b = healthy choanomastigote). The right photograph is of *C. bombi* following incubation at 50°C for 60 min, which shows a large proportion of ‘unhealthy’ cells (c = unhealthy amastigote, d = unhealthy choanomastigote).

Pilot experiments (Appendix S1) identified *C. bombi* cells that appeared less healthy compared to others. Based on pilot observations, criteria for these cells were specified (Table 1, Figure 2). To assess whether the number of healthy and unhealthy *C. bombi* cells differed between treatments, the number of healthy and unhealthy cells in the inoculum were counted using an improved Neubauer haemocytometer at ×400 magnification using a phase contrast microscope.

**TABLE 1:** Criteria for identifying healthy compared to unhealthy *Crithidia bombi* cells based on observations from pilot experiments.

Healthy	Unhealthy
Fast swimming choanomastigotes and promastigotes Larger amastigotes and choanomastigotes (estimated choanomastigote mean length is 6.03 ± 1.09 μm; Schmid‐Hempel & Tognazzo, 2010) Smoother surface texture of amastigotes and choanomastigotes Plump amastigotes and choanomastigotes Cells not burst Often more lifecycle stages present	Choanomastigotes and promastigotes that do not swim Smaller amastigotes and choanomastigotes Rougher surface texture of amastigotes and choanomastigotes Shrivelled amastigotes and choanomastigotes Multiple burst cells Choanomastigotes have flimsy tails Amastigotes are flatter and darker

#### 2.2.3 Inoculation

Individual workers were inoculated with a dose of 20,000 *C. bombi* cells. This dose was chosen to enable the assessment of prevalence and infection intensity, whilst maintaining field realism (Schmid‐Hempel & Schmid‐Hempel, 1993). Inoculation dose was also trialled in pilot experiments (see Appendix S1). On each day, two temperatures were tested, one for 10 min and one for 60 min. To prevent order effects, the order of temperatures was arranged such that a temperature was not tested twice on the same day and the order of temperatures was different for each incubation duration (Table S1). On each day, 32 individuals (four per colony) were inoculated per temperature and duration of incubation combination. Therefore, 320 individuals were inoculated in total, 64 each day.

Individuals were removed from their colonies and weighed in pre‐weighed vials to the nearest milligram (Scout SKX; Ohaus). Mass was used as a proxy for size since size can affect *C. bombi* infection intensity (Otterstatter & Thomson, 2006). Mass was used, rather than inter‐tegular distance or wing marginal cell length, due to time constraints on inoculation days. We appreciate that body mass may be influenced by sugar consumption, but as all bees had equal exposure to ad libitum food prior to weighing, this seems unlikely to have a meaningful impact on results. Bees were housed individually in nicot cages (Becky's bees), which are cylindrical containers adapted from hair rollers to house bees (see Figure S1). Bees were starved for 2 h prior to inoculation. Faeces were collected from 20 individuals per *C. bombi* stock colony and purified using a modified triangulation protocol (Baron et al., 2014; Cole, 1970). The triangulation protocol involves systematically centrifuging the faecal samples to remove contaminants, such as pollen. Cell concentration was calculated by viewing the purified faeces using an improved Neubauer haemocytometer under a phase contrast microscope at ×400. Purified faeces were mixed with sterile sugar solution (50% concentration) to produce a concentration of 20,000 cells in 30 μL. *Crithidia bombi* was suspended in sugar solution to mimic the transmission of *C. bombi* in nectar (Durrer & Schmid‐Hempel, 1994; Figueroa et al., 2019; but see Cisarovsky & Schmid‐Hempel, 2014). The inoculation solution was incubated in a PCR machine (Biometra TProfessional thermocycler, Jena Analytik, 070‐951) at the pre‐specified temperature for 10 or 60 min.

Immediately after incubation, the inoculum was given to adult worker bees. Individuals were given a 30‐μL droplet containing 20,000 cells. This volume was chosen because it is small enough for individual bees to drink in 4 h (H. S. Wolmuth‐Gordon, personal observation). The droplet was given to individuals in a 2‐mL syringe, which was attached to a hole in the base of the nicot cage (Figure S1; adapted from the OECD 247 protocol for Ecotoxicity testing). The end of syringes were removed to allow access to the inoculum. Individuals were left to drink for 4 h; if they had not consumed the entire droplet the individual was discarded from the experiment.

#### 2.2.4 Housing

Individuals were housed in nicot cages with sterile sugar solution (50% concentration) provided ad libitum via a 5‐mL syringe attached to the base of the nicot cage (see Figure S1). As before, the end of syringes was removed to allow access to the sugar. Syringes were replaced every 3 days to prevent fungal growth.

#### 2.2.5 Measuring infection

One week after inoculation bees were put in specimen tubes and approximately 10 μL faecal samples were taken using microcapillary tubes. Faecal samples were viewed under a phase contrast microscope at ×400. Prevalence and infection intensity were measured using an improved Neubauer haemocytometer (see Appendix S1). Prevalence was defined as the percentage of individuals infected with *C. bombi* and infection intensity the number of *C. bombi* cells in 1 μL of the faeces from one individual rounded to the nearest integer. The presence of each lifecycle stage of *C. bombi* (amastigote, choanomastigote and promastigote) and unhealthy cells (Table 1, Figure 2) were recorded. Prevalence, infection intensity and presence of lifecycle stages were measured once per individual.

### 2.3 Statistical analysis

Analyses were performed in RStudio “Prairie Trillium” (RStudio Team, 2022), R version 4.2.0 (R Core Team, 2022). All figures were created using the ggplot() function from the ggplot2 package (Wickham, 2016). For the majority of analyses, temperature and incubation duration were included as categorical variables. When analysing whether the presence of unhealthy cells was affected by the temperature and duration of incubation, temperature was included as a numerical variable because a quadratic term was required in the model. To test whether the number of unhealthy cells per microlitre of inoculum differed between treatments a generalised linear model with a quasi‐Poisson error distribution was used due to overdispersion. Temperature and incubation duration were included as fixed effects. The interaction between temperature and incubation duration was not included as this led to overdispersion.

To test whether the likelihood of infection varied with parasite incubation temperature and duration, a generalised linear model was constructed with a binomial error distribution and logit link. The full model included temperature, duration of incubation and their interaction as fixed effects. Bee body mass and colony were also included as covariates. For all analyses colony was included as a fixed effect rather than random effect due to the small number of individuals from each colony per treatment (Arnqvist, 2020; Gelman & Hill, 2006). A likelihood ratio Chi‐squared test and AIC values were used to compare reduced and full models. Model assumptions were checked graphically and using the DHARMa package (Hartig, 2022).

To investigate whether infection intensity was affected by parasite incubation temperature and duration a generalised linear model with a negative binomial error distribution was constructed using the glm.nb function from the MASS package (Ripley et al., 2022). Only infected bees were included in this analysis. Infection intensity was measured as cells per microlitre rounded to the nearest integer. In the full model, temperature, incubation duration and their interaction were included as fixed effects, in addition to bee mass and colony, which were included as covariates. Model fit was assessed as described above.

To test whether the presence of unhealthy cells was affected by the temperature and duration of incubation a generalised linear model with a binomial error distribution and logit link was used. In the full model, temperature, incubation duration and their interaction were included as fixed effects along with colony. Quadratic terms for temperature and the temperature interaction were added after plotting the model results.

To assess whether the presence of each lifecycle stage varied with incubation temperature and duration a separate generalised linear model, with a binomial error distribution and logit link, was used to analyse the presence of each lifecycle stage. The full model included temperature, incubation duration and their interaction and colony as fixed effects.

## 3 RESULTS

A total of 289 bees were successfully inoculated and screened for infection. Some individuals were lost from the sample because they did not drink the entire inoculum (*n =* 12), died before screening (*n =* 11) or did not defecate during screening (*n =* 8).

### 3.1 Do incubation temperature and duration affect the number of unhealthy cells in 1 μL of inoculum?

**FIGURE 3:**
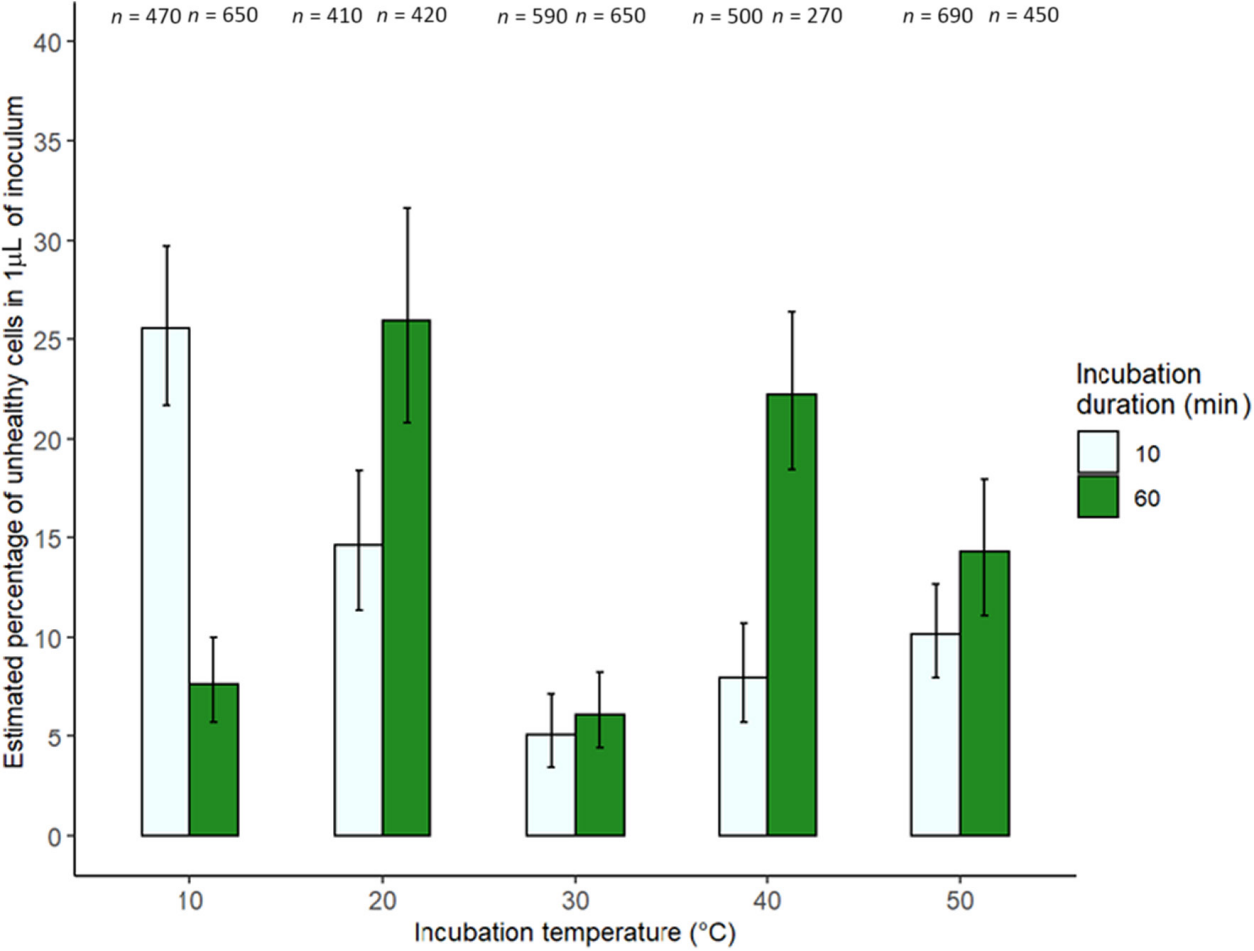
The estimated percentage of the number of unhealthy *Crithidia bombi* cells in 1 μL of inoculum following incubation at five temperatures for 10 (pale green, left) and 60 min (dark green, right). Error bars show 95% binomial confidence intervals and sample sizes of the total number of counted cells are above each bar.

Incubation temperature and duration did not affect the number of unhealthy cells in 1 μL of inoculum ($\chi_{4}^{2}$ = 39.4, *p* = .397; $\chi_{1}^{2}$ = 21.1, *p* = .619; Figure 3). Figure 3 shows the number of unhealthy cells as a percentage of the total number of counted cells in 1 μL. The number of unhealthy cells in 1 mL of inoculum was not analysed as a percentage due to model under dispersion.

### 3.2 Do incubation temperature and duration affect the prevalence of infection?

**FIGURE 4:**
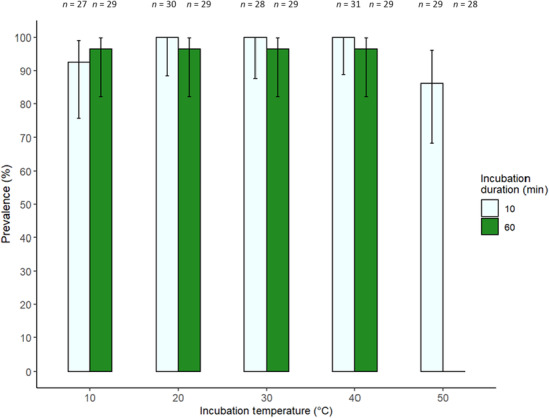
The prevalence of infection of *Crithidia bombi* in *Bombus terrestris audax* 1 week after infection. Prior to inoculation, *C. bombi* was exposed to five temperatures for two time periods. Pale green (left) indicates exposure to the temperature for 10 min and dark green (right) for 60 min. Error bars show 95% binomial confidence intervals. Sample sizes are given above each bar.

A model that removed colony and included temperature, incubation duration and their interaction, and bee mass had the best fit (see Appendix S1 for full model results). Prevalence of infection was not significantly affected by incubation temperature (*b* = 0.019, SE = 0.0405, *z* = 0.472, *p* = .637). However, incubation duration (*b* = 0.135, SE = 0.0479, *z* = 2.83, *p* = .00469) and the interaction between temperature and incubation duration significantly affected prevalence (*b* = −0.00445, SE = 0.00115, *z* = −3.86, *p* < .001; Figure 4). Between 20 and 40°C, longer incubation slightly reduced prevalence, however, at 50°C, longer incubation drastically reduced prevalence to 0%. Body mass of individual bees did not significantly affect prevalence (*b* = 3.5, SE = 2.12, *z* = 1.65, *p* = .0993; Figures S8 and S9).

### 3.3 Do incubation temperature and duration affect the intensity of infection in infected individuals?

**FIGURE 5:**
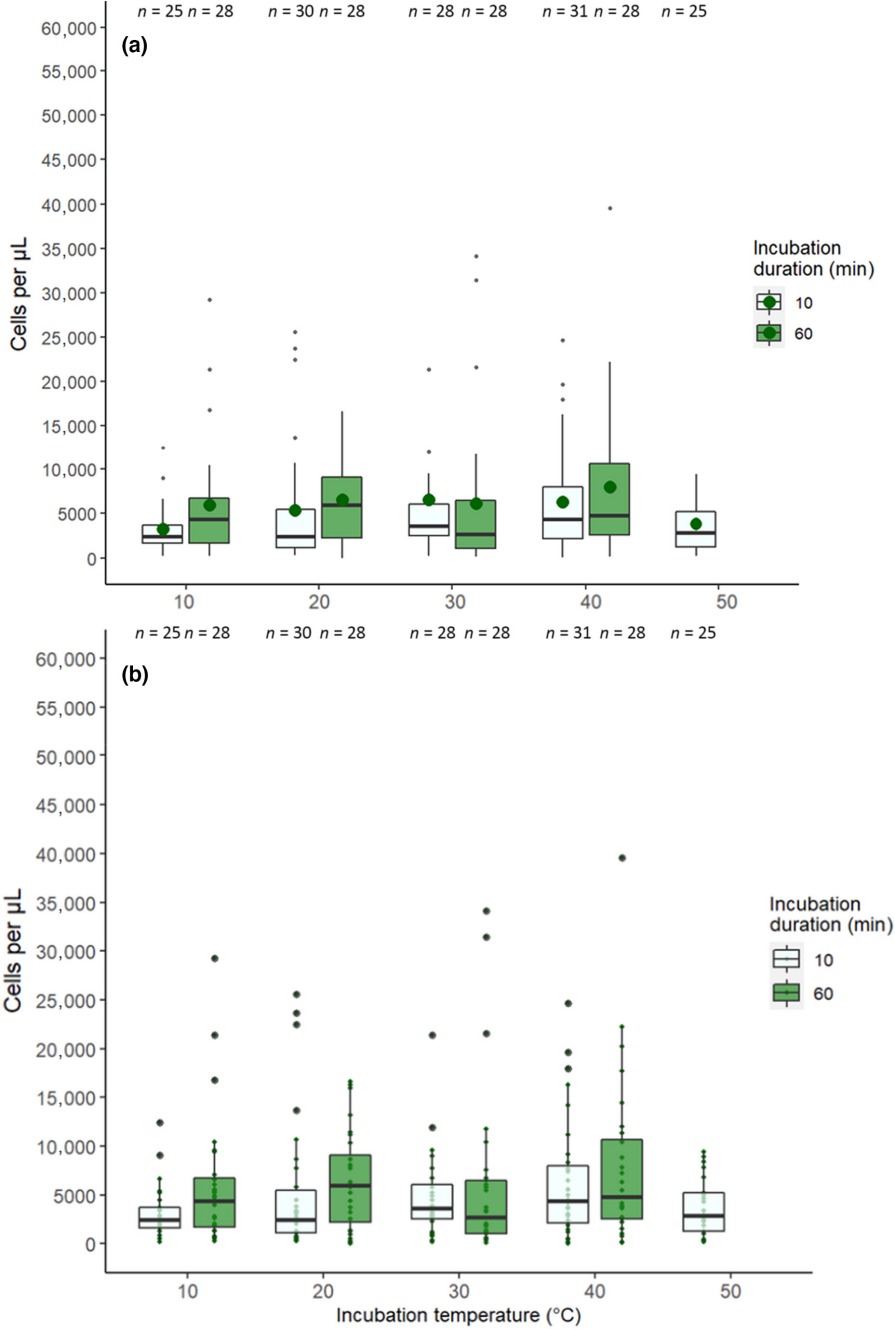
(a) Boxplot showing the infection intensity (cells per μL) of *Crithidia bombi* in *Bombus terrestris audax* 1 week after infection. Prior to inoculation, *C. bombi* was exposed to five temperatures for two time periods. Pale green boxplots (left) indicates exposure to the temperature for 10 min and dark green boxplots (right) for 60 min. Means are indicated by the large, dark green datapoints and sample sizes are above each boxplot. (b) Same as (a) without mean datapoints and including raw data as smaller datapoints.

A reduced model (including temperature, incubation duration as fixed effects, bee mass and colony as covariates) and excluding the interaction between temperature and incubation duration had the best fit. Temperature and incubation duration did not significantly affect infection intensity ($\chi_{4}^{2}$ = 311, *p =* .0785; $\chi_{1}^{2}$ = 308, *p =* .0837; Figure 5). Figure 5 shows that there was little difference in infection intensity between treatment groups. Furthermore, bee body mass and colony did not affect infection intensity ($\chi_{1}^{2}$ = 306, *p =* .0921; *χ*_7_ = 308, *p =* .0627).

### 3.4 Do incubation temperature and duration affect the presence of unhealthy *Crithidia bombi* cells in faeces?

**FIGURE 6:**
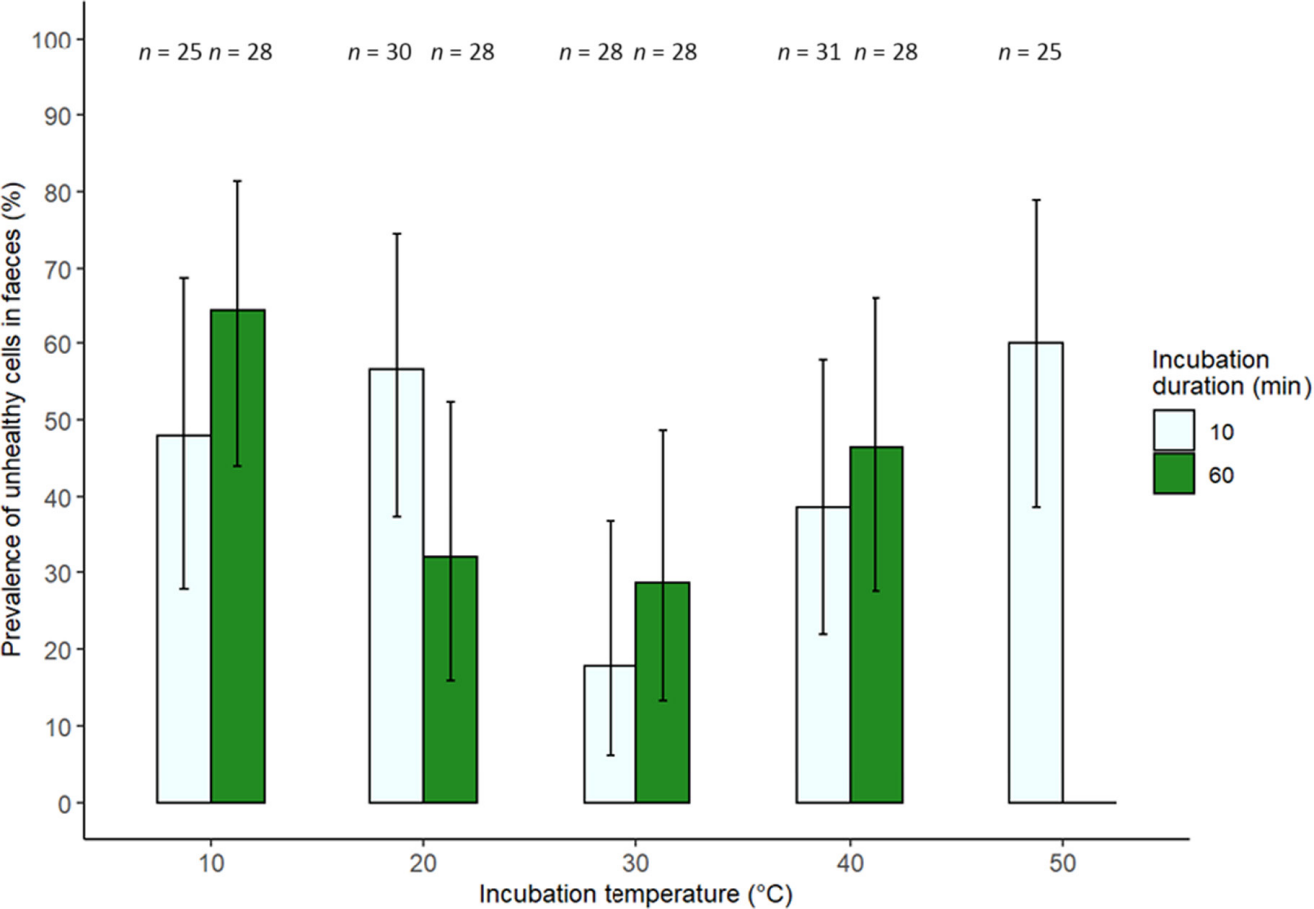
The prevalence of infected *Bombus terrestris audax* with unhealthy *Crithidia bombi* cells (based on criteria in Table 1 and Figure 2) 1 week after being infected with *C. bombi*. Prior to inoculation, *C. bombi* was exposed to five temperatures for 10 (pale green bars, left) or 60 min (dark green bars, right). Error bars show 95% binomial confidence intervals. Sample sizes are above each bar.

The best model included temperature, the interaction between temperature and incubation duration, the quadratic term for temperature and its interaction, incubation duration and colony. Temperature significantly affected the likelihood of observing unhealthy cells (linear term: *β* = 0.22, SE = 1.96, *z* = −2.26, *p* = .0236; quadratic term: *β* = 1.29, SE = 1.12, *z* = 2.32, *p* = .0206; Figure 6). After incubation for 10 min, the prevalence of burst cells was more than two times higher at 10°C compared to 30°C, and three times higher at 50°C compared to 30°C (Figure 6). A similar pattern was seen after incubation for 60 min, with the lowest prevalence of burst cells observed at 30°C. The interaction between temperature and incubation duration did not affect the prevalence of unhealthy cells (linear term: *β* = 0.239, SE = .239, *z* = −1.16, *p* = .248; quadratic term: *β* = 1.34, SE = 1.26, *z* = 1.25, *p* = .21). In addition, incubation duration and colony did not affect the chance of observing unhealthy cells (incubation duration: $\chi_{1}^{2}$ = 1.034, *p* = .309; colony: $\chi_{7}^{2}$ = 11.1, *p* = .133).

### 3.5 Do incubation temperature and duration affect the likelihood of observing the three *Crithidia bombi* lifecycle stages?

Amastigotes were present in 98% of infected individuals and choanomastigotes in 97.21% of infected individuals. Due to the very high prevalence of these lifecycle stages, the results were not further analysed. Promastigote lifecycle stages were present in 17.93% of infected individuals. Whilst the best model included temperature and incubation duration, neither temperature nor incubation duration significantly affected the presence of promastigotes ($\chi_{4}^{2}$ = 230, *p* = .203; $\chi_{1}^{2}$ = 229, *p* = .3).

## 4 DISCUSSION

Overall, we found that below 50°C, *C. bombi* prevalence was not affected by incubation temperature or duration, indicating that the parasite remains infectious across a wide range of temperatures. The interaction between incubation temperature and duration affected the prevalence of *C. bombi* at 50°C, resulting in no infections after 60 min. Furthermore, the intensity of infection was not affected by incubation duration or temperature. Despite being able to successfully infect and replicate once inside the host, unhealthy, burst *C. bombi* cells were more than twice as prevalent at 10°C compared to 30°C after incubation for 10 and 60 min, and more than three times as prevalent at 50°C compared to 30°C when incubated for 10 min. This suggests that temperature may cause physiological changes in the parasite, with the optimum temperature for *C. bombi* approximately 30°C. These changes appear to occur after infection because the incubation temperature and duration did not affect the presence of unhealthy cells in the inoculum.

The effect of incubation duration on prevalence of infection indicates that the duration of time *C. bombi* spends outside the host affects its ability to infect the next host. Between 20 and 40°C, incubating the inoculum for 60 min resulted in a ~4% decrease in prevalence at a given temperature, compared to incubating the inoculum for 10 min. This is a relatively small decrease when prevalence in worker populations in the field is high, for example 77.7% (Popp et al., 2012). However, towards the end of the summer prevalence tends to fall, for example, to 3.9% in August (Parsche & Lattorff, 2018), and at this time a 4% decrease would have a much larger effect on overall prevalence. In contrast to our results, Schmid‐Hempel et al. (1999) found a ~16% reduction in prevalence when *C. bombi* was left on a slide for 45 min prior to inoculation, compared to if used to infect hosts immediately. In addition, *C. bombi* left on a flower for 3 h exhibited reduced motility (Figueroa et al., 2019; Pinilla‐Gallego et al., 2022), which may affect its infectivity. We incubated *C. bombi* in solution, which suggests that reduced infectivity may not just be a product of it drying out in the environment, but rather other changes that occur when the parasite is outside the host. The larger effect seen by Schmid‐Hempel et al. (1999) indicates that in the field *C. bombi* may lose infectivity at a faster rate when deposited on petals compared to in nectar.

When considering the transmission of *C. bombi* in the field, the location of *C. bombi* on the floral structure needs to be considered. Studies have demonstrated that bees defecate in the middle of the flower into the nectar (Figueroa et al., 2019) and that *C. bombi* is transmitted via nectar (Durrer & Schmid‐Hempel, 1994). Sugar in the nectar may exert physiological stress on *C. bombi* as, Cisarovsky and Schmid‐Hempel (2014) found that *C. bombi* growth is inhibited at higher concentrations of sugar. This may have implications for *C. bombi* transmission in the field because the concentration of sugar in plant nectar varies between species (Pamminger et al., 2019). Consequently, *C. bombi* deposited in nectar in some plants with higher nectar sugar concentration may be under greater physiological stress. The likelihood of defecation by bumblebees on different parts of the flower can depend on the floral structure (Figueroa et al., 2019), indicating that the species and trait composition of plants in a landscape could influence the prevalence of *C. bombi*. Furthermore, when considering the duration of time that *C. bombi* is exposed to the environment, the rate of visitation by bumblebee foragers needs to be considered. The visitation rate of different plants varies with multiple floral design features, such as flower colour (Stanton, 1987) and the rate of nectar production (Mitchell, 1994). Therefore, plants with shorter visitation rates may be more likely to transmit *C. bombi*, particularly, for *C. bombi* cells shed on the surface of floral structures rather than in the nectar.

The response of *C. bombi* prevalence to temperature exposure appears not to be linear but a threshold response. The threshold for infectivity was surpassed at 50°C after 60 min, as no bees were successfully infected after this treatment. Lack of infectivity at higher temperatures could be due to the denaturation of enzymes and proteins (Copeland, 2000). The fact that infection intensity and the presence of the three lifecycle stages were not significantly affected by temperature shows that once established in a host *C. bombi* was able to successfully replicate, irrespective of its previous exposure. Temperature can affect the separate stages of parasite transmission to a host differently. Increased temperatures, for example, have been shown to increase the ability of the trematode, *Ribeiroia ondatrae*, to penetrate host skin, but reduces the ability of trematode larvae, called cercariae, to encyst after skin penetration (Paull et al., 2012). Furthermore, the increase in the proportion of unhealthy *C. bombi* cells at 50°C compared to 30°C indicates that morphological changes occurred following temperature exposure, but these changes were not visible immediately, because the presence of unhealthy cells in the inoculum did not vary between treatments. When exposed to higher temperatures another trypanosome, *Leishmania* spp, exhibits morphological changes to the cell structure and the parasite loses its ability to multiply (Zilberstein & Shapira, 1994). In *Leishmania* spp. these changes are reversible, and the parasite regains its ability to multiply when the temperature falls. This appears not to be true for *C. bombi* as exposure to 50°C was temporary. The lack of infectivity after prolonged exposure to 50°C suggests that *C. bombi* transmission may be curtailed in climates and seasons with very high temperatures. Interspecific and intercolony transmission may be lower in hotter areas of *C. bombi*'s range, such as in Spain (Jabal‐Uriel et al., 2017), South America (Gallot‐Lavallée et al., 2016; Schmid‐Hempel et al., 2014) and parts of North America (Cordes et al., 2012). Furthermore, due to climate change, temperatures above 40°C are becoming increasingly common and more prolonged (Coumou et al., 2013). For example, in the summer of 2022 temperatures surpassed 40°C in the United Kingdom (Kendon, 2022). Consequently, *C. bombi* is expected to be exposed to temperatures above 40°C more frequently than previously.

However, before drawing conclusions on the effects of temperature on transmission, the impacts of temperature on the host need to be understood. For example, the infectivity of *C. bombi* may decrease at higher temperatures, but the susceptibility of hosts may also change at high temperatures. Whilst we did not examine this, previous work has shown varying effects of temperature on host susceptibility to *C. bombi* (Palmer‐Young et al., 2019; Tobin et al., 2019). Tobin et al. (2019) concluded that incubating hosts at 21–29°C had no effect on prevalence or infection intensity. When Palmer‐Young et al. (2019) tested a wider range of temperatures, infection intensity of *C. bombi* declined by 81% when hosts were incubated at 37°C compared to 21°C. These studies were conducted in the lab, in a non‐stressful environment with food ad libitum, whilst, in the field, food may be less available. Nutritionally stressed bees exhibit higher mortality when infected with *C. bombi* (Brown et al., 2000), and therefore, the effects of temperature on host susceptibility may be different in the field. However, a more important consideration for understanding whether the response of the parasite to high temperatures is relevant for transmission is host behaviour. At temperatures above 24°C, bumblebee flight ability decreases (Kenna et al., 2021) and at extremely high temperatures, such as above 40°C, bumblebees may reach their thermal maximum and enter heat stupor (Martinet et al., 2021). If bumblebees are unable to fly at temperatures above 40°C, transmission will not be affected by reduced *C. bombi* infectivity because the bumblebees will not encounter the parasite. However, thermal tolerance varies between species, with some Mediterranean sub‐species, such as *B. xanthopus*, able to withstand extended periods of time at these temperatures (Martinet et al., 2021; Oyen et al., 2016). It is unclear whether these species can fly over 40°C or whether they are just more resistant to heat stupor (Martinet et al., 2021). If they can fly, extreme temperatures may reduce pathogen transmission to heat‐tolerant species.

In contrast, at lower temperatures, *C. bombi* infection ability was not impeded, despite a higher proportion of cells looking unhealthy at 10°C compared to 30°C. The fact that *C. bombi* infectivity was approximately constant between 10 and 40°C suggests that temperature does not play a major role in the seasonal dynamics of *C. bombi* prevalence, which peaks in early summer in the northern hemisphere (Parsche & Lattorff, 2018; Popp et al., 2012). Based on our results *C. bombi* is likely equally as infective to emerging spring queens at 10°C as it is to foraging workers in the peak of summer. Rather, population demographic changes as the summer progresses likely play a major role in seasonal peaks in prevalence (Parsche & Lattorff, 2018; Popp et al., 2012). However, seasonal fluctuations in prevalence have only been studied in limited parts of *C. bombi's* range, so it would be interesting to test whether seasonal patterns in prevalence are the same across hotter regions, where temperatures surpass 40°C.

Finally, here we only looked at the effect of temperature on infectivity, whereas, when outside the host, *C. bombi* may be exposed to varying levels of humidity, UV radiation and potentially precipitation. These factors may also affect infectivity in isolation or may interact to alter infectivity. Climatic factors other than temperature, have been shown to affect the prevalence of some bumblebee pathogens including *Vairmorpha bombi* (Manlik et al., 2023; McNeil et al., 2020), *C. bombi* and *C. expoeki* (Ivers et al., 2022). Specifically, the prevalence of *V. bombi* can be affected by temperature, humidity, precipitation and cloud cover (Manlik et al., 2023). In addition, some have found a positive correlation between *V. bombi* prevalence and spring precipitation (McNeil et al., 2020). It is likely that UV radiation affects *C. bombi* survival because *C. bombi* survives for shorter time periods on sunny compared to shaded flowers (Figueroa et al., 2019). Sunlight‐level UV radiation has been shown to increase the mortality of nematode larvae (van Dijk et al., 2009), emphasising the importance of considering all aspects of climate variability. Exposure to different environmental variables whilst *C. bombi* is on flowers compared to when on nest material may mean *C. bombi* is more transmissible in one environment compared to the other. For example, if UV radiation reduces *C. bombi* survival, transmission on flowers may be lower than transmission from contaminated nest material within the colony at the same temperature.

## 5 CONCLUSIONS

In conclusion, our study showed that the parasite, *C. bombi*, was less infective the longer it spent outside the host under experimental conditions. This may suggest that the likelihood of transmission in the wild is higher on flowers with shorter visitation times. In addition, the infectivity of the parasite was not affected by exposure to temperatures between 10 and 40°C. *Crithidia bombi* was no longer infective after exposure to 50°C for 60 min. However, it is unlikely that many bumblebee species would fly and, therefore, encounter the parasite at this temperature. Infection intensity was not affected by temperature, however, extreme temperatures appear to induce morphological changes to *C. bombi* cells in the faeces of infected individuals. We investigated the effect of one climate variable on parasite infectivity when in reality multiple climate factors will vary in the environment. Assessing the effect of these on hosts and parasites in isolation, and when interacting, will further our understanding of the epidemiology of host–parasite interactions across different climates and help to predict the effects of climate change in the future.

## AUTHOR CONTRIBUTIONS

**Hannah S. Wolmuth‐Gordon:** Conceptualization (lead); data curation (lead); formal analysis (lead); funding acquisition (equal); investigation (lead); methodology (equal); project administration (lead); writing – original draft (lead); writing – review and editing (lead). **Mark J. F. Brown:** Conceptualization (equal); funding acquisition (equal); methodology (equal); supervision (lead); writing – review and editing (supporting).

## ACKNOWLEDGEMENTS

None.

## FUNDING INFORMATION

This work was funded by a Royal Holloway University of London Scholarship.

## CONFLICT OF INTEREST STATEMENT

We declare no conflicts of interest.

## Supporting information


Appendix S1.

## Data Availability

DOI for data: http://doi.org/10.5281/zenodo.7760010.
