# Supplementary material for: Transmission of a bumblebee parasite is robust despite parasite exposure to extreme temperatures
Source: Ecol Evol. 2023 Jul 25;13(7):e10379. doi: 10.1002/ece3.10379 (PMC10368942; doi:10.1002/ece3.10379)
Supplement: Supplementary file 1 — Appendix S1. [file ECE3-13-e10379-s001.docx]

**Supplementary Material**

**Table 1** Blocking design for the order of incubation duration and temperatures tested on each day to prevent order effects.

| **Incubation duration (mins)** | **Temperature (° C)** | | | | |
| --- | --- | --- | --- | --- | --- |
|  | **Day 1** | **2** | **3** | **4** | **5** |
| 10 | 20 | 30 | 10 | 50 | 40 |
| 60 | 30 | 40 | 50 | 20 | 10 |


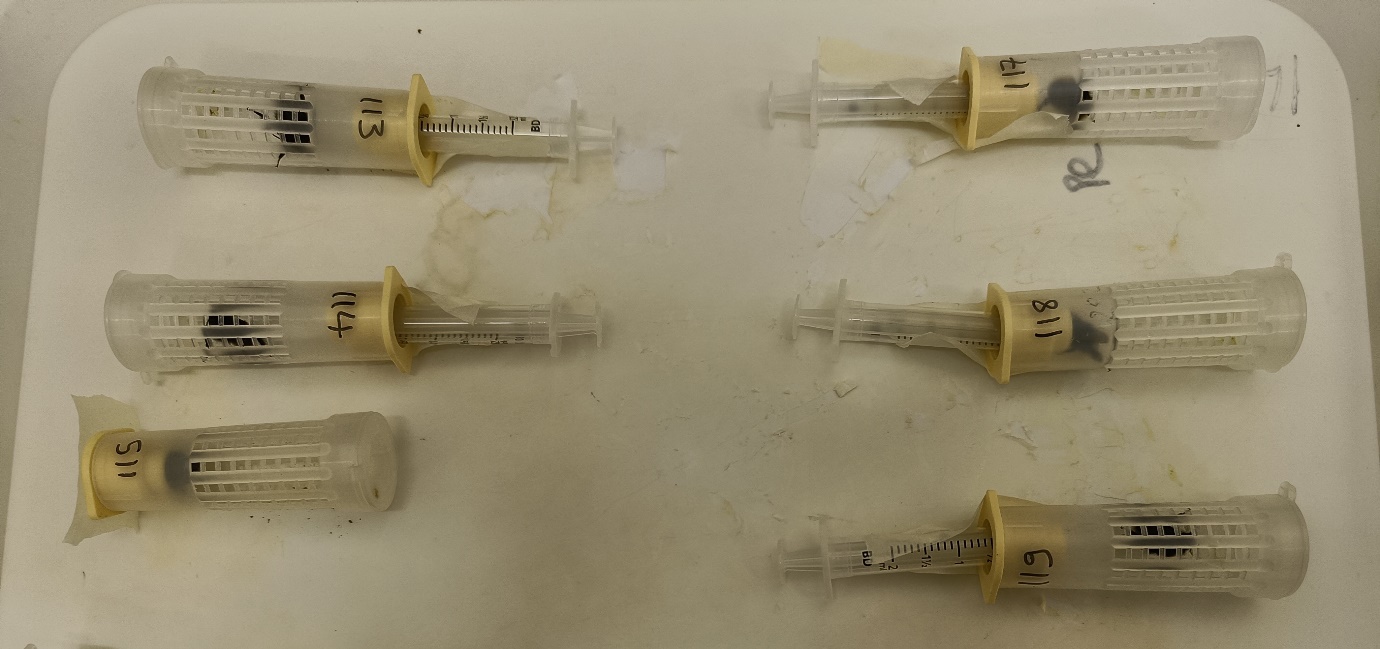


**Fig. 1** *Bombus terrestris audax* were housed in nicot cages (Becky’s bees, UK) throughout the experiment. Here, bees are being inoculated using 2ml syringes attached to the base of the nicot cage with masking tape. After inoculation, 2ml syringes were replaced with 5ml syringes containing sterile sugar solution (50% concentration) provided *ad-libitum*. Syringes were replaced every three days to prevent fungal growth.

**Measuring infection**

The first author conducted the experiment alone and therefore, it was not possible to be 100% blind to the treatment being counted. However, the bees were given individual ID numbers and more than one treatment was tested each day. When measuring infection one week later the first author could only see the ID number of each bee and not which treatment it was from. Since a large number of bees were inoculated it was essentially impossible to remember which ID number was from each treatment and therefore, counting was effectively, but not formally, blind.

**Pilot experiment 1**

A pilot experiment was conducted to determine the inoculation dose, and temperature and incubation duration ranges. The same protocol was followed as outlined in the methods section. Two *Bombus terrestris audax* colonies, with 85-150 workers were ordered from Agralan (UK). *Crithidia bombi* (please see Materials and Methods in the Main text for details of origin) was exposed to 10°C and 40°C for 10 and 60 minutes. 32 individuals (16 per colony) were used in each temperature, incubation duration combination. An inoculation dose of 15,000 cells was trialled to maximise the chance of infection whilst, also enabling the effect of treatment on prevalence to be investigated.

Results

**Table 2.** Number of bees lost from the sample and the reason why.

| **Reason bees were lost from sample** | **Frequency** |
| --- | --- |
| Failed to drink inoculum | 4 |
| Died before screening | 3 |
| Failed to defecate during screening | 1 |


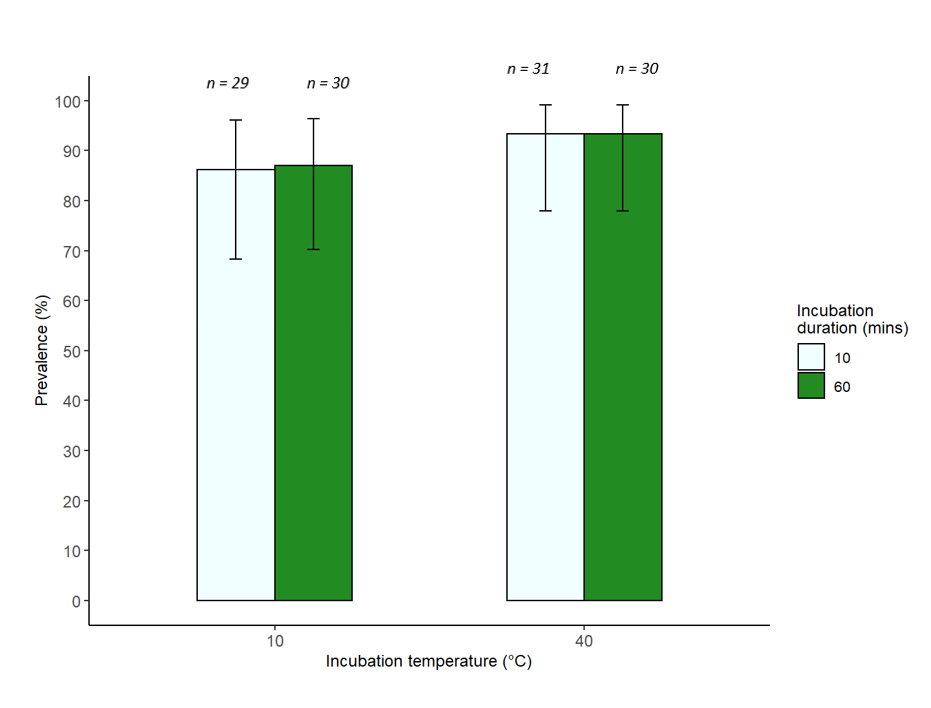


**Fig. 2** Prevalence of *C. bombi* in bees one week after pilot inoculation of 15,000 cells. *Crithidia bombi* was exposed to two temperatures for 10 (pale green, left) and 60 minutes (dark green, right) prior to inoculation. Error bars are 95% binomial confidence intervals.


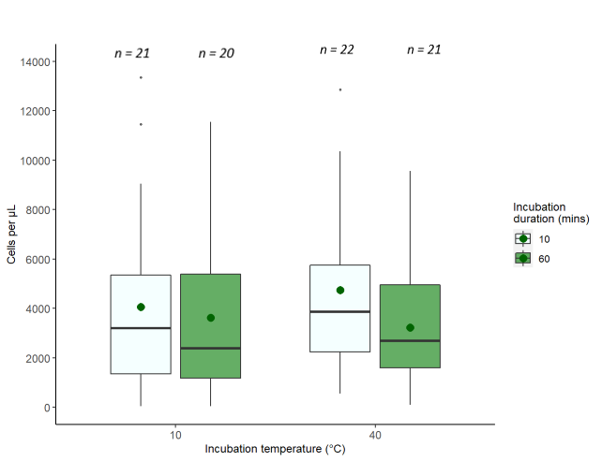


**Fig. 3** Boxplot showing the intensity of infection of *C. bombi* in bees one week after a pilot inoculation of 15,000 cells. *Crithidia bombi* was exposed to two temperatures for 10 (pale green, left) and 60 minutes (dark green, right) prior to inoculation. Means are indicated by the large, dark green datapoints and sample sizes are given above.

**Pilot experiment 2**

The prevalence was higher than expected in pilot experiment 1. A lower inoculation dose of 8,000 cells was trialled to lower the rate of infection and enable us to test the effect of temperature and incubation duration of prevalence. Furthermore, a higher maximum temperature of 50°C was tested. The inoculum was incubated at these temperatures for 10 minutes. 60 minutes was not tested at 50°C because it was assumed that the rate of infection would be lower than for 10 minutes at 50°C. 16 bees were tested in each treatment group, 8 from each colony.

Results

**Table 2** Number of bees lost from the sample and the reason why.

| **Reason bees were lost from sample** | **Frequency** |
| --- | --- |
| Failed to drink inoculum | 2 |
| Died before screening | 1 |
| Failed to defecate during screening | 1 |


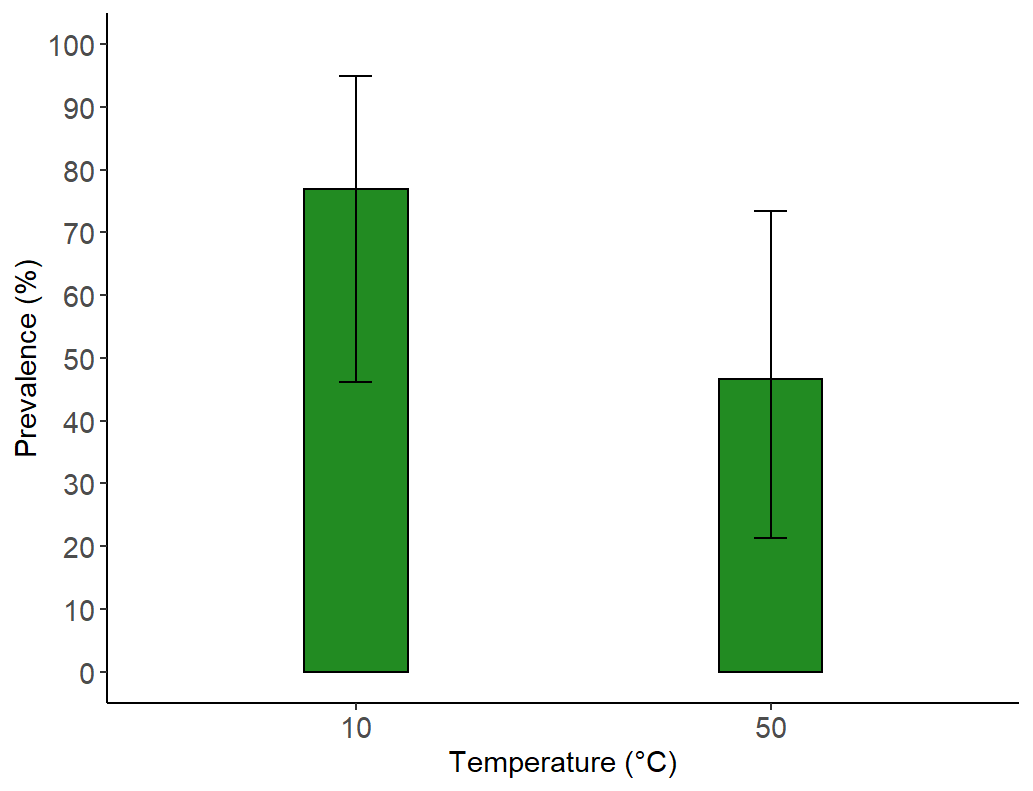

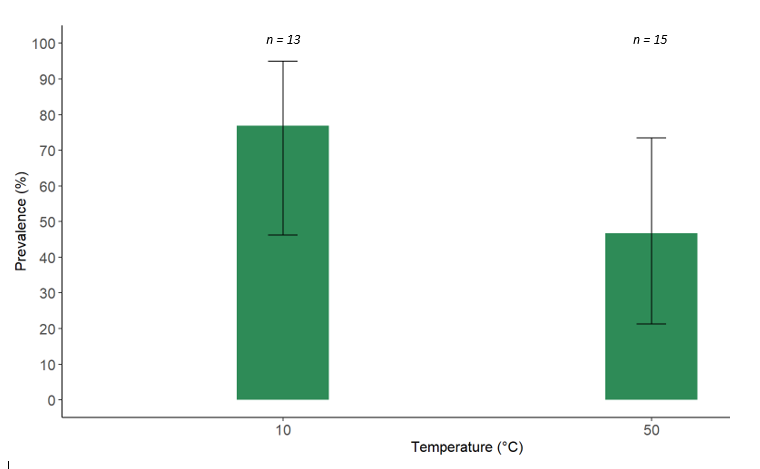

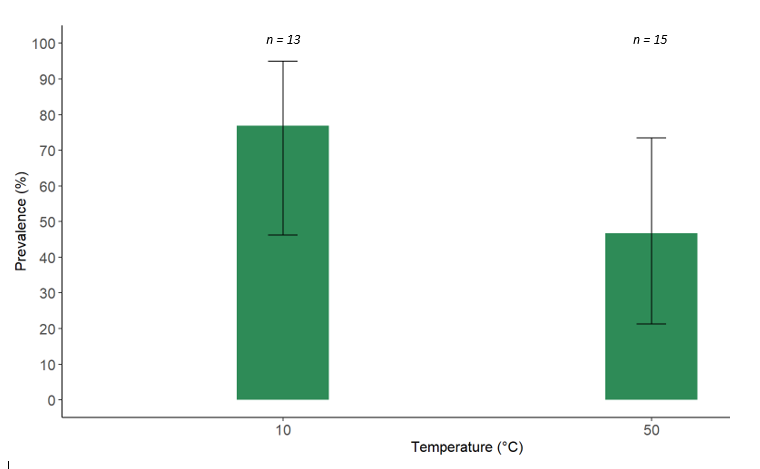


**Fig. 4** Prevalence of *C. bombi* in bees one week after pilot inoculation of 8,000 cells. *Crithidia bombi* was exposed to two temperatures for 10 minutes prior to inoculation. Error bars are 95% binomial confidence intervals. Sample sizes given above.


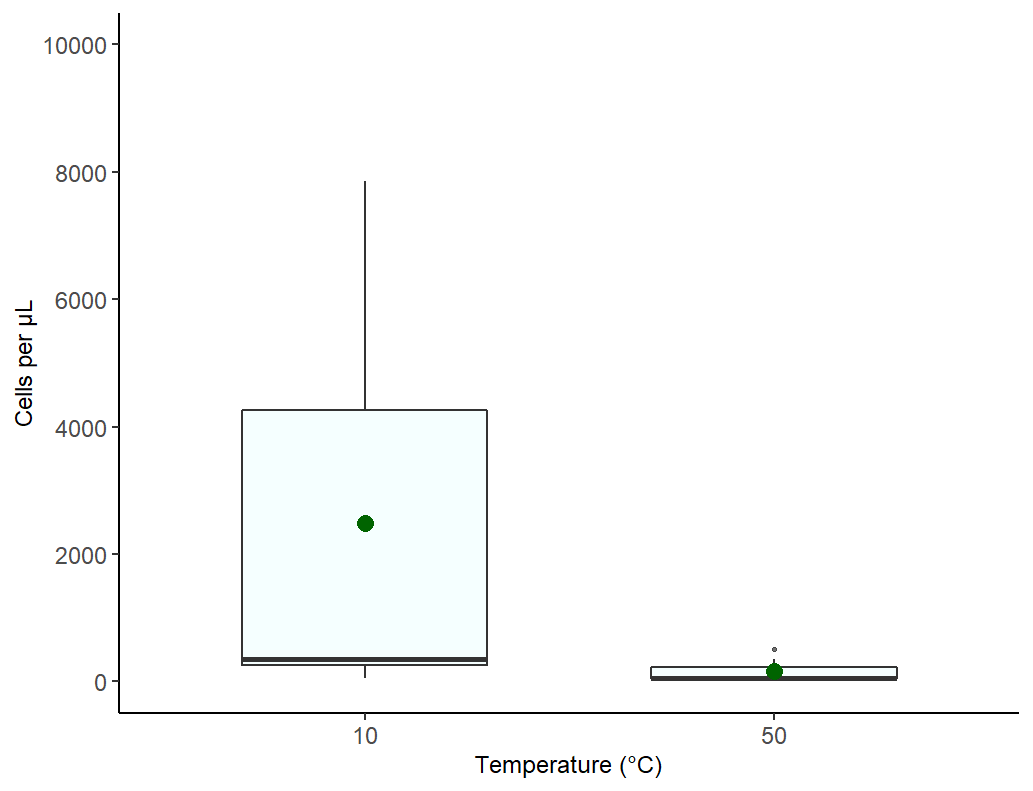

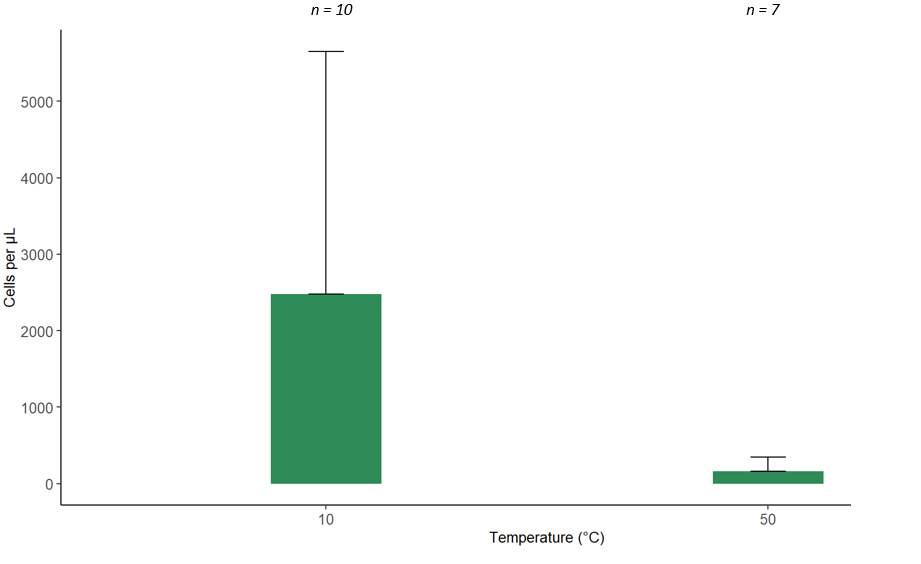

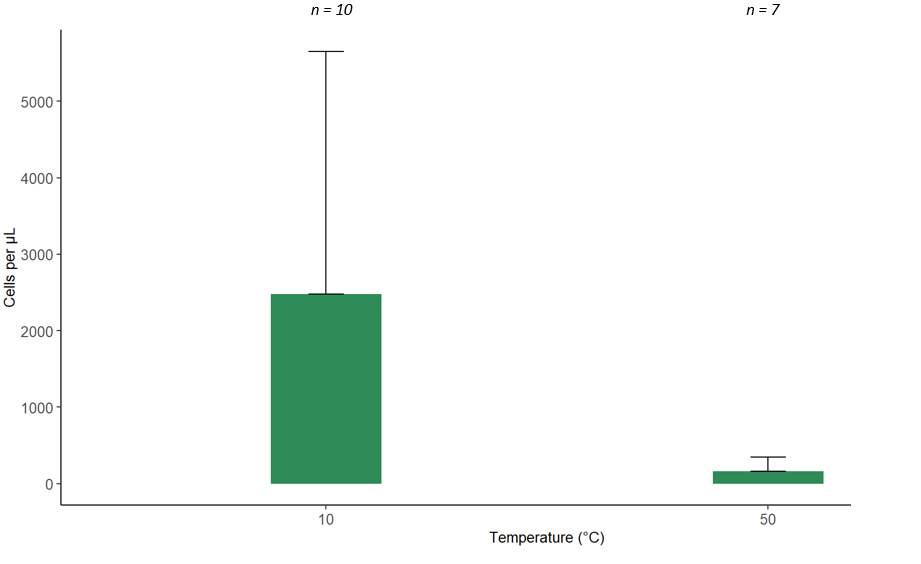


**Fig. 5** Boxplot showing the infection intensity of *C. bombi* in bees one week after a pilot inoculation of 8,000 cells. *Crithidia bombi* was exposed to two temperatures for 10 minutes prior to inoculation. Means are indicated by the large, dark green datapoints and the sample sizes are given above.

**Experiment 1. Inoculation dose of 8,000 cells**

This experiment was conducted using a dose of 8,000 cells, based on results from the pilot experiments. After observing the prevalence, we decided to increase the inoculation dose to 20,000 as the prevalence was not as expected given previous data and the pilot results.

| **Reason bees were lost from sample** | **Count** |
| --- | --- |
| Failed to drink inoculum | 20 |
| Died | 4 |
| Failed to defecate | 5 |


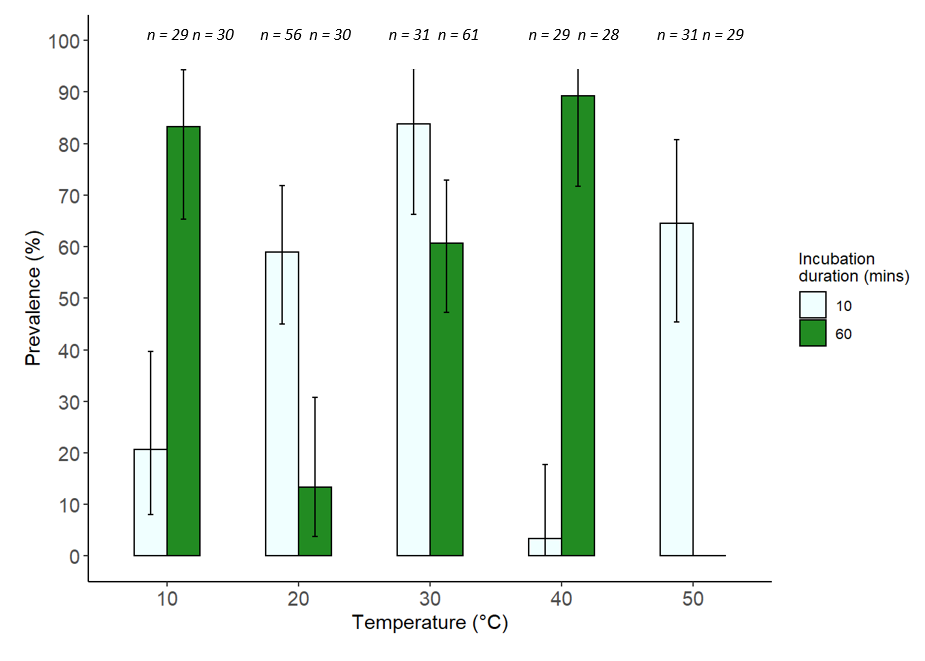


**Fig. 6** The prevalence of infection of *C. bombi* in individuals one week after infection with 8,000 cells. Prior to inoculation, *C. bombi* was exposed to five temperatures for two time periods. Pale green (left) indicates exposure to the temperature for 10 minutes and dark green (right) for 60 minutes. Error bars show binomial 95% confidence intervals. Sample sizes are above the bars.


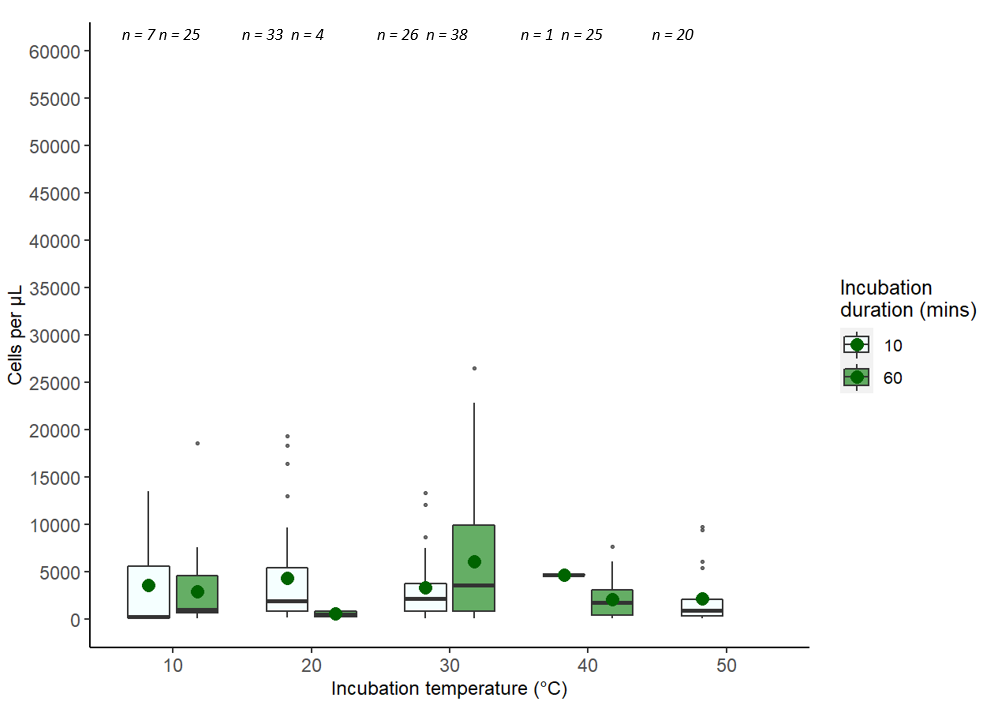


**b)**

**a)**


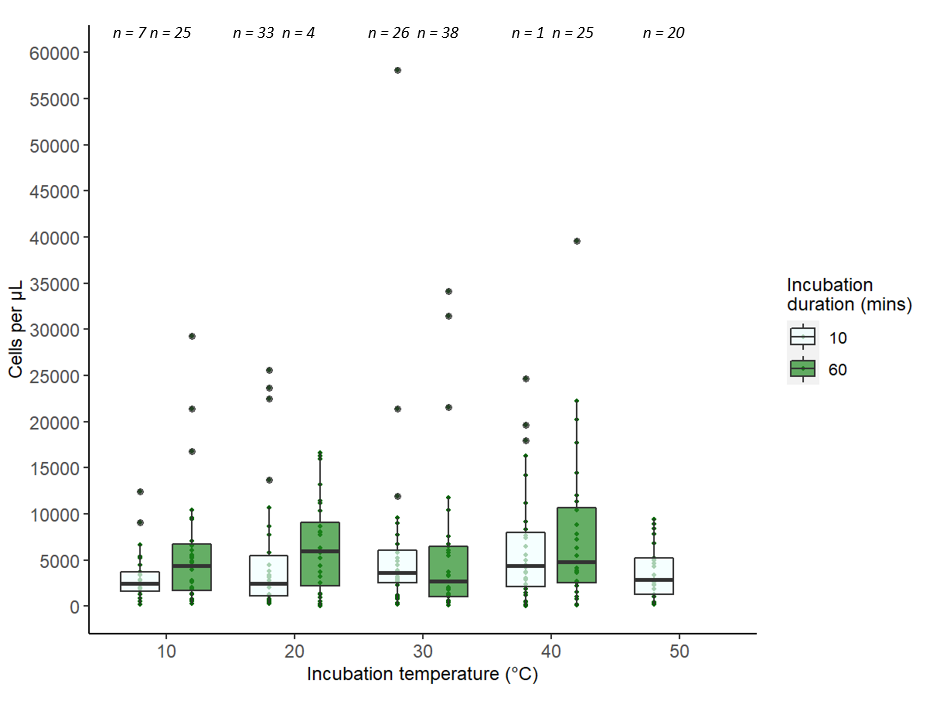


Fig. 7 a Boxplot showing the infection intensity (cells per μL) of *C. bombi* in individuals one week after infection with a dose of 8,000 cells. Prior to inoculation *C. bombi* was exposed to five temperatures for two time periods. Pale green (left) indicates exposure to the temperature for 10 minutes and dark green (right) for 60 minutes. Means are indicated by the large, dark green datapoints and sample sizes are given above. **b)** Same as a) without mean datapoints and including raw data as smaller datapoints.

**Experiment 2. Inoculation with 20,000 (experiment in manuscript)**

**Do incubation temperature and duration affect the prevalence of infection?**

In the full model (including temperature, incubation duration and their interaction, bee mass and colony), prevalence of infection was significantly affected by incubation temperature (X^2^_4_ = 132, *p >* 0.001), incubation duration (X^2^_1_ = 89.6, *p* > 0.001) and their interaction (X^2^_4_ = 52.953, *p* > 0.001; Fig. 3). Body mass of individual bees and colony did not significantly affect prevalence (X^2^_1_ = 89.0, *p =* 0.424; X_7_ = 76.3, *p =* 0.0808; Fig. 8 and Fig. 9 Supplementary material). This model was not presented in the main analyses because colony was insignificant and resulted in a worse fitting model.


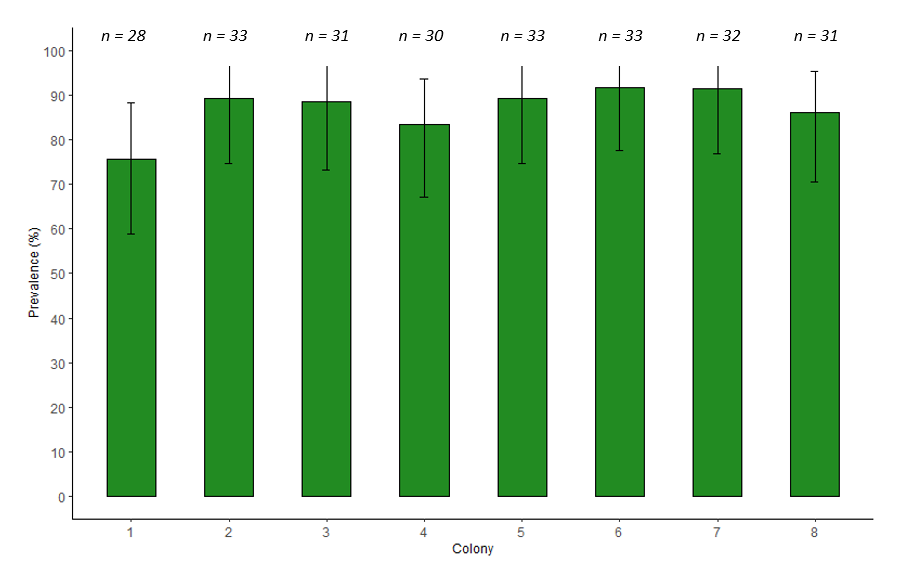


**Fig. 8 P**revalence of *C. bombi* in individuals from each colony after inoculation with 20,000 cells. Error bars show 95% confidence intervals. Samples sizes are given above bars.


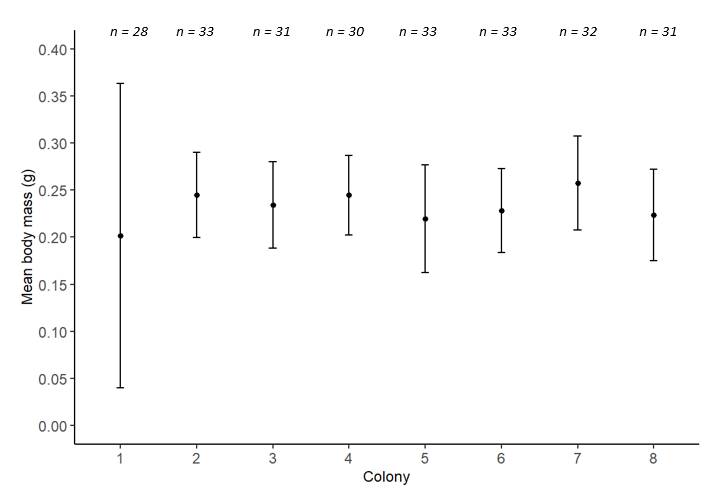


**Fig. 9** Mean body mass of bees from each colony before inoculation with *C. bombi.* Error bars show upper and lower standard deviations and sample sizes are given above the points.

**
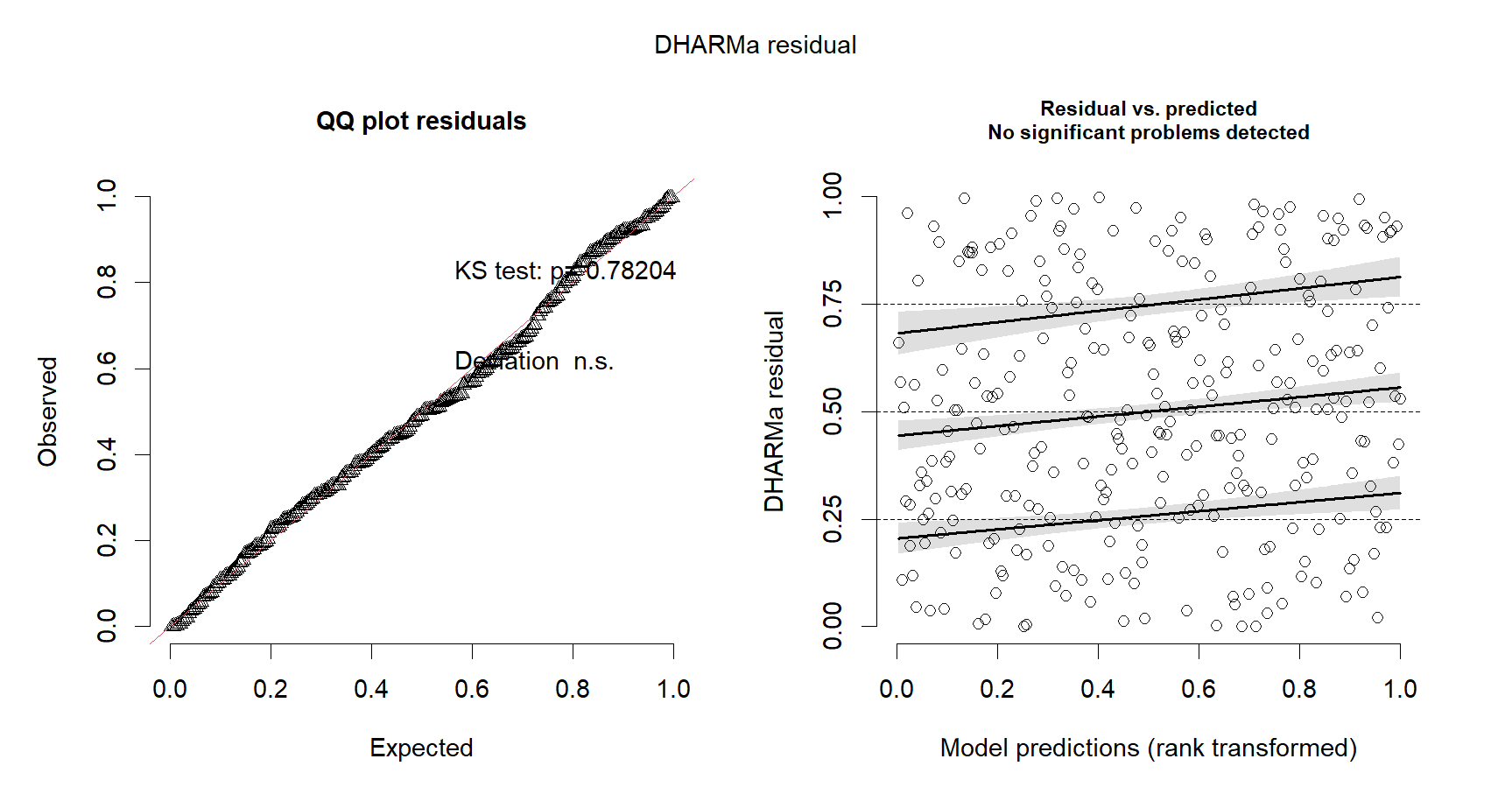
**
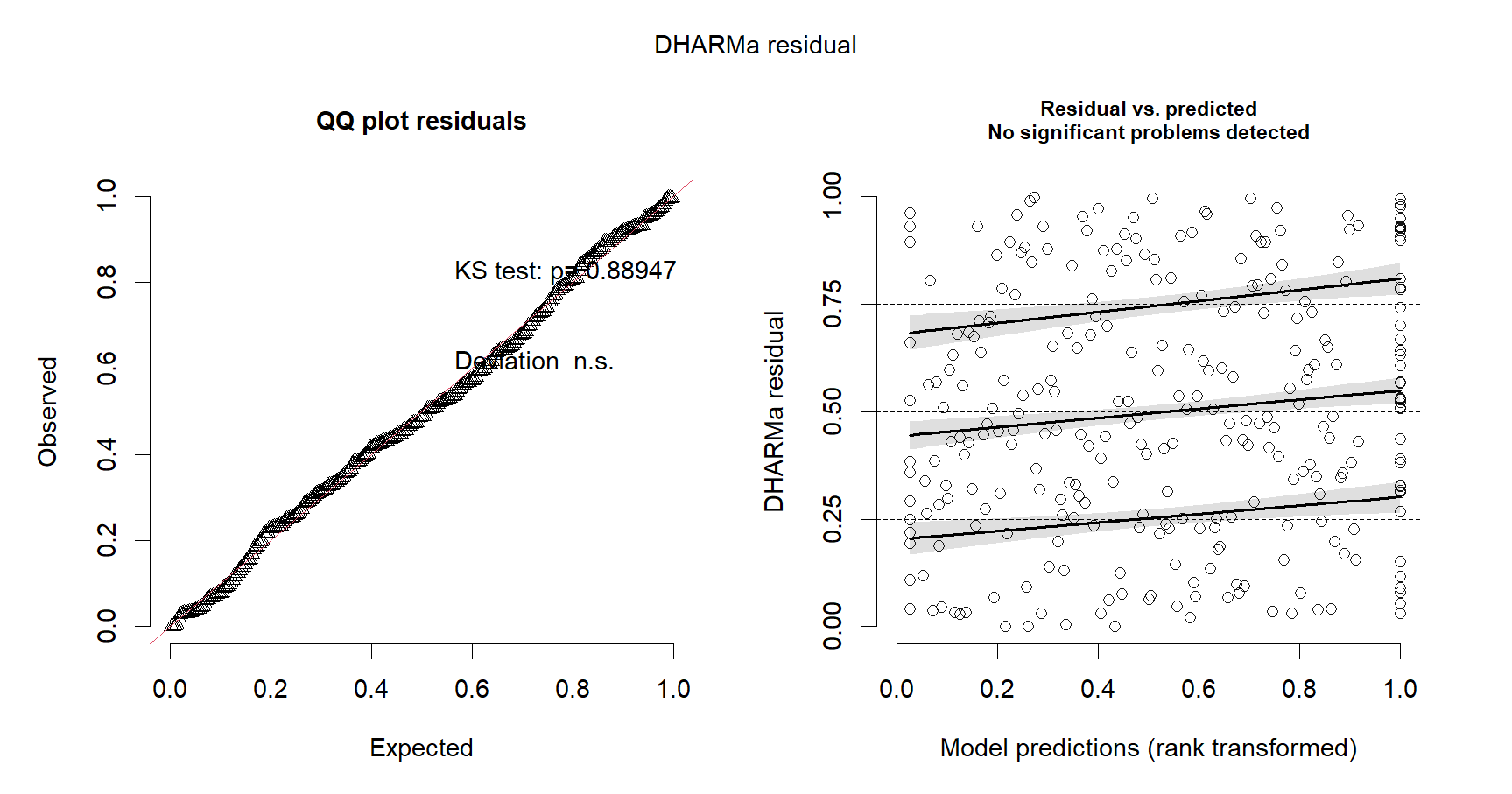


b)

a)

**Fig. 10** Residual plots for checking assumptions of (a) the full model and (b) the reduced model without colony, using the package “DHARMa” (Hartig, 2022)
